# Validation of energy intake recorded by a 7-day pre-coded food diary against measured energy expenditure in a group of Norwegian adults

**DOI:** 10.1371/journal.pone.0215638

**Published:** 2019-04-18

**Authors:** Anne Marte Wetting Johansen, Jannicke Borch Myhre, Anette Hjartåker, Lene Frost Andersen

**Affiliations:** Department of Nutrition, Institute of Basic Medical Sciences, University of Oslo, Oslo, Norway; Universidade Federal do Rio Grande do Sul Instituto de Ciencias Basicas da Saude, BRAZIL

## Abstract

The aim of this study was to validate energy intake (EI) reported by a pre-coded food diary (PFD) against energy expenditure (EE) measured by the ActiReg system consisting of an activity and position monitor and a calculation program (ActiCalc). Dietary intake was recorded by the PFD and EE was measured by the ActiReg system over a 7-day period. One hundred and twenty adult participants completed the study, 42 men and 78 women. The average group EI was 17% lower compared to measured EE. The 95% limits of agreement were 6.7 and -2.9 MJ/day. Of all participants, 68% were classified as acceptable reporters, 29% as underreporters and only 3% as overreporters. Fifty percent of the men and 30% of the women were classified into the same quartile for EI and EE, whereas 5% of both men and women were ranked in the opposite quartile by the two methods (weighted kappa coefficient = 0.29). Pearson correlation coefficient between reported EI and measured EE was 0.49 (p<0.001). High BMI was related to larger underreporting when EE was low. Furthermore, this study found that PFD underestimates EI on the group level with an average of 17% and showed large variation in the validity of the PFD on the individual level.

## Introduction

For several decades nutritional researches have worked on improving dietary assessment methods. A valid dietary assessment method measures the true dietary intake over a defined time period. Errors in diet-report instruments can result in important diet-disease relationships being overlooked [1]. For instance, Bingham et al. suggested that dietary measurement error might explain the absence of a significant association between dietary fat intake and breast cancer risk in cohort studies [2]. Underreporting of energy intake (EI) is a common phenomenon in nutrition research [3]. Since EI is closely linked to the intake of other nutrients, underreporting of EI typically results in underreporting of nutrient intakes. Underreporting of EI is either due to under-eating, underrecording or a combination of the two. Factors that have been associated with underreporting are obesity, weight consciousness, restrained eating, socio-economic status and social desirability [4, 5].

Studies using recovery biomarkers show that dietary assessment methods like food records and recalls give more accurate nutrient intake estimates compared to the commonly used food frequency questionnaire [6]. A pre-coded food diary (PFD) was developed at the Department of Nutrition at the University of Oslo, Norway in 2000, for use in a nationwide survey among children and adolescents (UNGKOST 2000) [7]. Later, a section of alcoholic beverages and a few more variants of coffee were included in the PFD to make it suitable for use among adults. Since the year 2000, the PFD has been used in many studies in Norway [8–14]. In order to enrol a sufficient number of participants and to minimize dropout in future surveys, it is important to develop dietary assessment methods that in addition to being accurate, decrease the workload of the participants to a minimum. The PFD takes about 10–15 minutes per day to fill out and is relatively easy to complete [15]. Compared to weighed records, the PFD requires little writing, and estimation of portions is simplified by the use of either pre-coded household measures or pictures of portion sizes. Furthermore, a pre-coded food diary decreases the workload related to post-study data processing compared to methods involving manual coding of foods.

The doubly labelled water (DLW) method is regarded as an excellent biomarker of EE [16], but the high expenses and need for special instruments make the method less useful in validation studies with relatively large samples. The ActiReg system is a far more cost-effective way of measuring EE. The method has previously been validated against DLW among women and against indirect calorimetry among a group of adults, with acceptable results [17]. The aim of the present study was to validate EI assessed by a 7 day PFD against EE measured by the ActiReg system among a group of adults in Norway.

## Methods

### Subjects

The subjects were recruited by posters, e-mails and personal requests at fitness centers and workplaces in the Oslo area, and included students and employees at the University of Oslo, friends and family of employees of the University of Oslo, students at Oslo University College, and employees from the Norwegian army and from the Norwegian Food Safety Authority. Since the participants were a convenience sample, the participation rate is not known. Due to limitations of the ActiReg, participants who took part in swimming or strength exercise more than 3 times per week were not included in the study. A total of 170 men and women volunteered to participate in the study. Thirty-eight persons dropped out (22%) and 12 participants (9%) were excluded due to incomplete EE measurements. A total of 120 subjects, aged 19 to 69 years completed the study, 42 men and 78 women. Participants who wore the ActiReg less than 3 days were excluded from the analysis. Of the included participants, 102 participants wore the ActiReg for 7 days, 12 wore it for 6 days, 3 for 5 days, 1 for 4 days and 2 participants wore the Actireg for 3 days. The study was approved by the Regional Committees for Medical and Health Research Ethics, Region South (reference number S-02132) and by the Norwegian Center for Research Data (reference number 200200623). Written informed consent was obtained from all participants.

### Design

The recruitment was carried out from 2002 to 2004. A nutritionist gave the participants oral instructions of the methods in small groups or one-to-one. Prior to the study start the participants’ height and weight were measured. Recording of food intake and measurement of activity level were conducted during the same 7 days. The participants were instructed to maintain their normal eating habits throughout the registration period. The study also included a relative validation of the PFD by use of 7-days weighed food records one week after the recording in the PFD [18]. Letters with individual data on EI, nutrient intake and EE were sent to each of the participants after the study was finished.

### Food diary and photographic booklet

The 18 pages PFD comprises 294 pre-coded food items grouped together according to a typical Norwegian meal pattern. The design of the PFD is similar to a cross-table with food listed on the left and time span across the top. Each day is divided into five time spans, of which four time spans cover four hours each, e.g. from 06:00 to 10:00, from 10:00 to 14:00 etc., and one time span covers eight hours from 22:00 to 06:00. The first pages contain cold and hot beverages, bread, spread and cereals. The middle section contains typical dinner dishes, followed by dessert/cakes, fruit, snacks/candy and supplements at the end of the PFD. Each food group is supplemented with open-ended boxes where consumption of items or dishes not covered by the PFD can be described. For the majority of food items and beverages, amounts are presented in predefined household measures (cups, slices, spoons, etc.). For about 25% of the food items and beverages, there is a box to fill in portion size as shown in a corresponding photographic booklet. The photographic booklet contains 15 colour picture series; one with two pictures, one with tree pictures and thirteen with four pictures showing different portion sizes ranging from small to large [15]. The participants were instructed to fill in the PFD immediately after each meal was finished and foods eaten outside the home were registered in the PFD at the end of the day.

Dietary data were entered by scanning, using the Teleform program (6.0) (Datascan, Oslo, Norway). Foods in the open-ended boxes were manually coded and added to the data file. Daily intake of foods, energy and nutrients were calculated using an in-house food database and software system (KBS, version 3.1, 2002) developed at the Department of Nutrition, University of Oslo. KBS is based on the official Norwegian Food Composition Table, and is continuously supplemented with data on new food items and nutrients.

### The ActiReg system

The participants in our study had their activity pattern registered continually for 7 days except during sleep and during showering and other water activities. The ActiReg system (PreMed AS, Oslo, Norway) uses a combined second-to-second recording of body position and motion to calculate EE. The monitor has two pairs of position and motion sensors connected by cables to a battery-operated storage unit fixed to a waist belt. Each pair of sensors is attached by medical tape to the chest and the front of the right thigh, respectively. The storage capacity of Actireg is sufficient to cover more than 30 days of continuous registration of normal activity. In total, there are 16 codes for different combinations of body positions and presence/absence of movement in the two sensors together. The data is transferred to a computer and the codes are converted by the program ActiCalc, which uses calculation models based on literature values for the energy cost of various activities. Further details about the ActiReg monitor are described elsewhere [17]. The ActiReg has previously (both alone and in combination with heart rate) been validated against indirect calorimetry and DLW [17]. The results showed no significant differences in mean EE between the ActiReg and the reference method. The correlation coefficients between EE measured with ActiReg and calorimetry and DLW were 0.76 and 0.45, respectively [17].

### Weight, height, basal metabolic rate and physical activity level

Body weight and height were measured out in the field by project staff. Participants were non-fasting, wearing light clothes and no shoes. Weight was measured using a digital scale (± 0.1 kg), and height was measured to the nearest cm. Body mass index (BMI) was calculated from measured weight and height (kg/m2). Basal metabolic rate (BMR) was derived from equations based on age and gender. Physical activity level (PAL) was measured by the ActiReg system.

### Statistical analysis

Data analysis was performed using SPSS for Windows release 24.0 (SPSS, Inc., Chicago, IL, USA). Results are presented as means and standard deviations (SD). Differences between means were tested using paired t-test and were considered to be statistically significant at p<0.05. Pearson correlation coefficients were calculated to evaluate the linear correlation of recorded EI and measured EE at the individual level. Individuals were classified into quartiles, and method agreement is expressed as the proportion of participants classified into the same, adjacent, and extreme quartile by the two methods. The weighted kappa coefficient was calculated to measure the agreement between the methods regarding classification into quartiles. The ratio EI:EE expressed the accuracy of reported EI. As suggested by Black for seven days of diet records [19], acceptable reporters (ARs) were defined to be in the EI:EE range of 0.76–1.24, whereas underreporters (URs) and overreporters (ORs) were defined as having a ratio less than 0.76 and larger than 1.24, respectively.

Independent samples t-test was used to evaluate possible differences in anthropometric data and percentage underreporting between groups. The visual agreement between the methods is presented as proposed by Bland and Altman using a plot of the difference between the two methods against the average of the measurements [20]. This type of plot shows the magnitude of disagreement, outliers and systematic errors associated with the test method.

Low EE was defined as values equal to or below the median, whereas high EE was defined as values above the median, i.e. 13.30 MJ/day for men and 10.15 MJ/day for women.

## Results

Characteristics of the participants and the main results are presented in Table 1. Sixty-five percent of participants were women and mean (SD) age and BMI were 35 (13) years and 24.1 (3.4) kg/m^2^ among all participants. Mean age and BMI did not differ significantly between men and women. EE was significantly higher than EI for the group of participants as a whole (mean difference 1.9 MJ/day, p<0.01). The difference between EE and EI was significantly larger for men (mean difference 2.8 MJ/day) than for women (1.4 MJ/day, p<0.01). When EE-EI was calculated as the percentage of EE, the gender difference was no longer statistically significant (20.3% versus 13.3%, p = 0.07).

**Table 1:** Participant characteristics (mean and SD) and main results.

	All (n = 120)	Men (n = 42)	Women (n = 78)
Age (y)	35 (13)	34 (12)	36 (13)
Height (cm)	172 (9)	180 (8)	168 (6)
Weight (kg)	71.8 (12.9)	80.9 (12.4)	66.9 (10.3)
BMI (kg/m^2^)	24.1 (3.4)	24.9 (3.3)	23.7 (3.3)
BMR (MJ/day)	6.6 (1.0)	7.8 (0.7)	6.0 (0.5)
EI (MJ/day)	9.4 (2.4)	10.8 (2.7)	8.7 (2.0)
EE (MJ/day)	11.3 (2.5)	13.6 (1.8)	10.1 (1.3)
EE-EI (MJ/day)	1.9 (2.4)	2.8 (2.7)	1.4 (2.0)**
EI:EE	0.84 (0.20)	0.80 (0.19)	0.87 (0.21)
PAL	1.71 (0.19)	1.76 (0.22)	1.68 (0.16)
Pearson r EE and EI	0.49**	0.32*	0.26*

BMI, Body Mass Index; BMR, Basal Metabolic Rate; EI, energy intake; EE, energy expenditure; PAL, physical activity level; EE:BMR

* p<0.05

** p<0.01 (Independent samples t-test)

Pearson correlation coefficient between EE and EI for all participants was 0.49 (p<0.001), whereas gender-specific correlations were weaker; 0.32 (p = 0.04) for men and 0.26 (p = 0.02) for women. Overweight participants had a higher correlation (r = 0.63, p<0.001) between EE and EI as compared to normal weight persons (r = 0.40, p<0.01).

Fifty percent of the men and 30% of the women were classified into the same quartile for EI and EE, while 5% of both men and women were ranked in the opposite quartile by the two methods (weighted kappa coefficient = 0.29).

Table 2 presents results for the relationship between EE and percentage underreporting. The participants with high EE, underreported on average their EI with 21%, whereas participants with low EE underreported with approximately 11%. Among those with low EE, the subgroup with high BMI (≥ 25 kg/m2) underreported significantly more than the subgroup with BMI < 25 kg/m2 (24.5% vs. 7.3%, p = 0.025). However, among those with high EE, there was no significant difference in percentage underreporting between normal weight and overweight participants.

**Table 2:** Percentage underreporting in relation to energy expenditure (EE) and body mass index (BMI).

Categories of EE and BMI	n	Percentage underreporting ((EE-EI)/EE)x100	p-value^2^
All participants			
Low EE^1^ (≤ median MJ/d)	62	10.6	<0.01
High EE^1^ (> median MJ/d)	58	21.2	
Low EE^1^ (≤ median MJ/d)			
BMI < 25	50	7.3	0.03
BMI ≥ 25	12	24.5	
High EE^1^ (> median MJ/d)			
BMI < 25	29	19.7	0.52
BMI ≥ 25	29	22.7	
BMI < 25			
Low EE^1^ (≤ median MJ/d)	50	7.3	<0.01
High EE^1^ (> median MJ/d)	29	19.7	

MJ/d; megajoule/day.

^1^ Cut-offs for high and low EE was calculated from median EE; 13.30 MJ/day for men and 10.15 MJ/day for women

^2^ Differences between groups were tested with Independent Samples T-test

**Fig 1:**
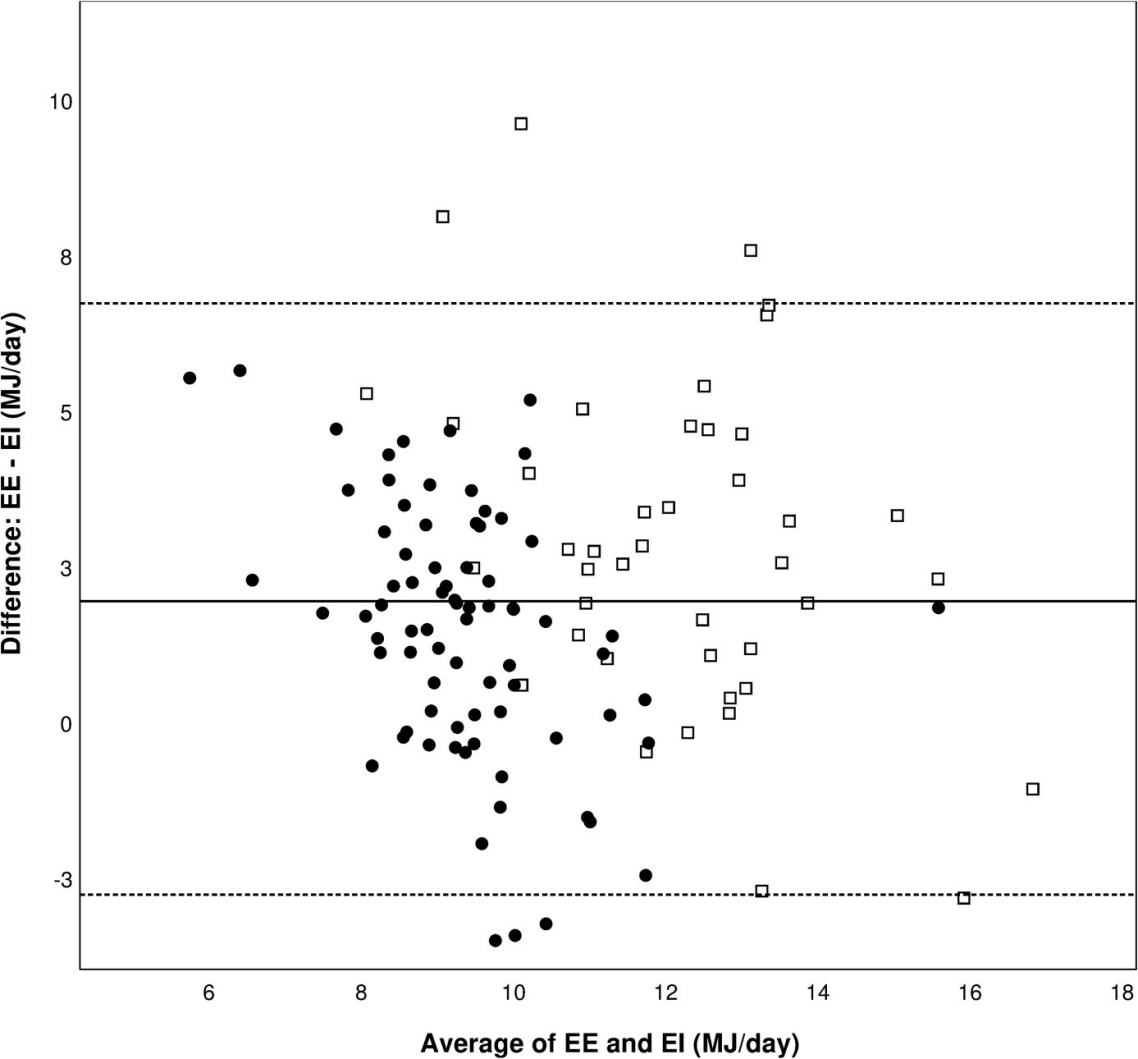
Bland-Altman plot of the difference between measured energy expenditure (EE) and estimated energy intake (EI) plotted against the mean of EE and EI for men (□) and women (●). The solid line indicates the mean difference between the methods while the dashed lines indicate ±1.96 SDs. MJ, mega joule.

Fig 1 is a plot of the difference between EE from the ActiReg and EI from the PFD against the average of the measurements. The plot illustrates that both underreporting and overreporting of EI occurred, of which underreporting was most prominent. The 95% confidence limits of agreement varied from 6.7 MJ/day to −2.9 MJ/day, which indicates wide discrepancies between the two methods at the individual level.

**Fig 2:**
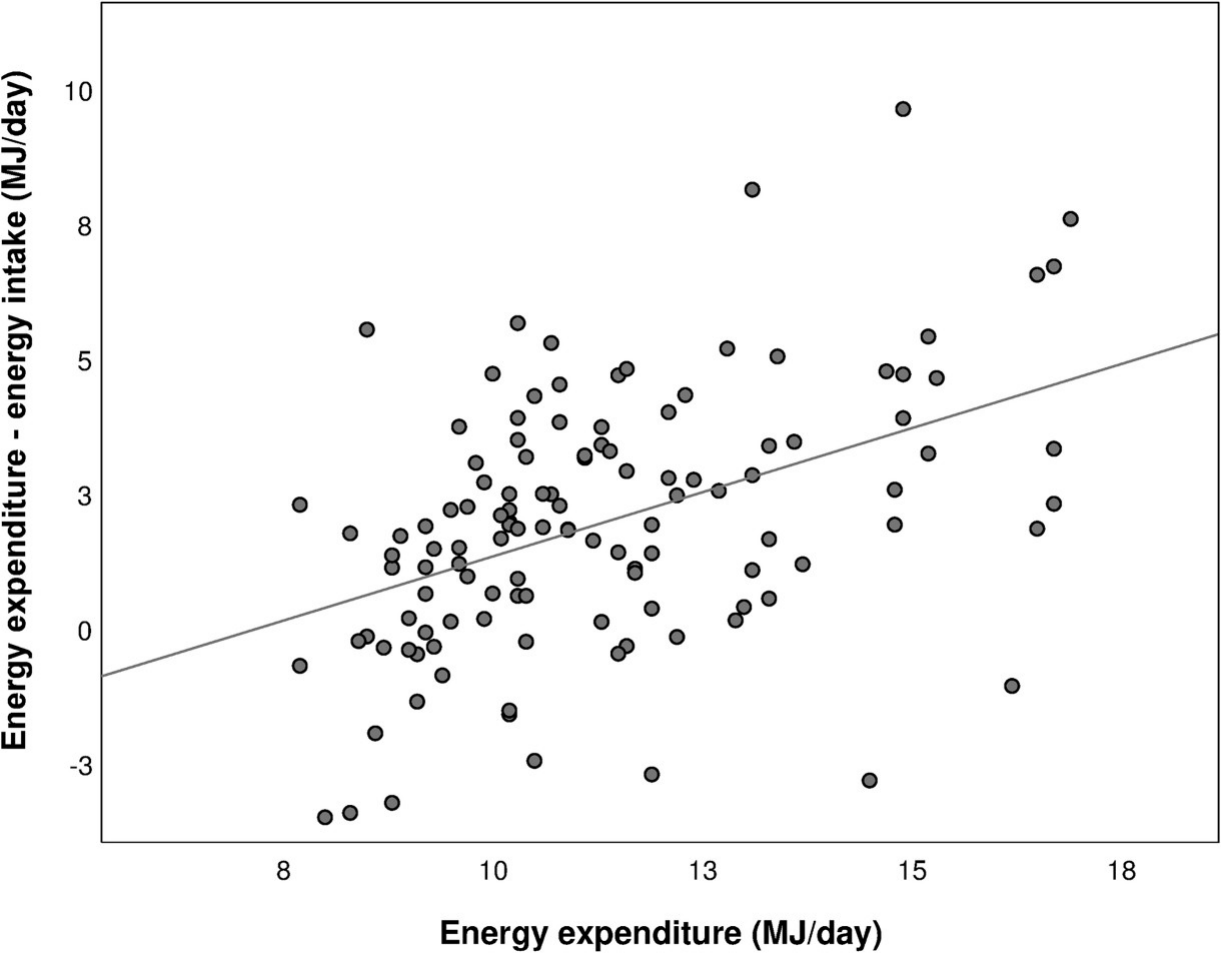
Correlation between underreporting of energy intake (energy expenditure—energy intake) and energy expenditure. Pearson r = 0.45 (p<0.01). MJ; megajoule.

Fig 2 shows the correlation between underreporting, expressed as EE–EI, and EE. The magnitude of underreporting increased significantly with increasing EE (r = 0.45, p<0.01).

Table 3 presents characteristics of all acceptable reporters (ARs) and underreporters (URs) as well as stratified by gender. Sixty-seven percent of the men and 72% of the women in our study were classified as ARs, whereas 33% of the men and 27% of the women were classified as URs. Three women (4%) were classified as overreporters (data not presented in Table 3). When comparing all ARs and URs, the EI was significantly lower among URs, whereas BMI and EE were significantly higher among URs.

**Table 3:** Characteristics (mean and SD) of acceptable reporters (ARs) and underreporters (URs).

	All (n = 117)		Men (n = 42)		Women (n = 75)	
Variable	ARs (n = 82)	URs (n = 35)	ARs (n = 28)	URs (n = 14)	ARs (n = 54)	URs (n = 21)
Age (y)	34 (13)	38 (12)	32 (10)	38 (14)	36 (14)	38 (12)
Height (cm)	173 (9)	173 (8)	180 (8)	180 (6)	168 (6)	168 (5)
Weight (kg)	70.5 (12.5)	76.1 (13.2)*	78.2 (12.6)	86.4 (10.3)*	66.5 (10.4)	69.3 (10.2)
BMI (kg/m^2^)	23.6 (3.2)	25.4 (3.4)**	24.0 (3.1)	26.6 (3.2)*	23.4 (3.3)	24.6 (3.4)
BMR (MJ/day)	6.5 (1.0)	6.8 (1.1)	7.7 (0.8)	8.0 (0.6)*	6.0 (0.5)	6.1 (0.5)
EI (MJ/day)	10.1 (2.2)	7.5 (1.9) **	11.8 (2.2)	8.6 (2.1)**	9.3 (1.5)	6.6 (1.5) **
EE (MJ/day)	11.1 (2.1)	12.1 (2.4)*	13.2 (1.7)	14.4 (1.9)	10.0 (1.3)	10.6 (1.1)
EE-EI (MJ/day)	1.0 (1.5)	4.6 (1.6) **	1.4 (1.7)	5.8 (1.7) **	0.8 (1.3)	3.9 (0.9) **
Pearson r EE and EI	0.77**	0.76**	0.66**	0.65*	0.62**	0.63**

BMI, Body Mass Index; BMR, Basal Metabolic Rate; EI, energy intake; EE, energy expenditure

*p<0.05

**p<0.01 (Independent samples t-test)

## Discussion

Results from the present study showed that the PFD underreported EI by an average of 17% compared to EE measured with the ActiReg system. Participants with high EE underreported more than participants with low EE, independent of BMI. Among participants with low EE, high BMI was associated with larger underreporting.

The PDF has previously been validated against measured EE in children, adolescents and older men [11, 15, 21]. Nine-year-olds and 13-year-olds underreported EI by an average of 18 and 24–34%, respectively [15, 21]. In a group of older men the mean difference between EE and EI was –0.2 MJ/day (95% CI: -1.5, 1.1) in normal weight participants and -2.4 MJ/day (95% CI: -3.4, -1.4) in overweight/obese participants [11]. Based on these findings, Stea et al. concluded that the PFD was suitable for estimating EI among normal weight individuals, but not for overweight or obese individuals. A similar validation study of a 7-day PFD against ActiReg among adults in Denmark found lower mean underreporting (12%) than in the present study [22]. This could be due to lower overall participant burden, a less health-conscious group of participants, greater variation of portion sizes in the Danish study, or a combination of these factors. Mean measured EE did not seem to differ between the Danish study and the present study. A review of different dietary assessment methods validated against EE measured by DLW, showed a similar extent of EI underreporting in studies of estimated records as the present study (mean ± SD EI:EE of 0.84 ± 0.10) [23].

The correlation between EI and EE is regarded acceptable (Pearson r = 0.49), and slightly lower than what was found in the Danish validation study referred to above (r = 0.56) [22]. According to definitions by Lombard et al., a correlation coefficient between 0.20 and 0.49 is regarded as acceptable, whereas one above 0.50 is regarded as good [24]. Among children and adolescents in Norway, the correlation coefficient between EI and EE varied from 0.28 (9-year-olds) to 0.47–0.74 (two studies of 13-year-olds) [15, 21].

The PFD’s ability to classify individuals into the same quartile as the reference method was good among men (50%), and acceptable among women (30%) [24]. Only 5% of both men and women were classified in the opposite quartile. The weighted kappa coefficient was 0.29, which is regarded acceptable [24]. The wide scattering of the individual differences between the methods clearly shows great variation in reporting accuracy (Fig 1). The plot shows that underreporting was much more prominent than overreporting.

The ActiReg has previously been validated against DLW, and the results showed that ActiReg overestimated mean EE by 0.4 MJ/day [17], suggesting that the difference we found between EE and EI might be overestimated. Possibly, the awareness of wearing measurement equipment could have led to increased physical activity during the study period. Thirteen-year-olds increased their level of physical activity when wearing the ActiReg in a similar validation study of the PFD [21]. If the activity level is increased during the recording period either to please the researchers or to lose weight, without a simultaneous increase in EI, the EE:EI ratio will increase. The Nordic Nutritional Recommendations (NNR) 2012 has published values for energy requirements, based on gender, age groups and level of physical activity [25]. The NNR reference value for the age group 30–60 years and high activity level (PAL = 1.80) is 12.4 and 9.9 MJ/day for men and women, respectively. In our study sample, we found an EE of 14.1 MJ/day for men and 9.9 MJ/day for women over 30 years. For normal weight men over 30 years (n = 10), measured EE was 14.3 MJ/day. This shows that on average, male participants in our study had a particularly high EE, which was either due to an unusually high habitual activity level, and/or a temporary increase in activity level above the habitual level during the study period.

Previous studies suggest a negative association between social desirability and reporting accuracy [4, 26]. Subjects who tend to under-eat or under-record due to social desirability may also be more active for the same reason, resulting in a wider gap between reported EI and measured EE. The participants in our study received their individual results on EI, nutrient intake and EE, which might have pushed them towards wanting to make a better impression, i.e. eating less and/or healthier and/or exercising more.

The reported EI was significantly lower than measured EE and was considerably lower than expected from the reference value for energy requirement referred to above, an indication of either underreporting, under-eating or a combination. If body weight had been measured not only before but also after the food intake registration week, we could have known more about the role of dieting among the study participants during the study. As shown in Table 3, the difference between EE and EI is 4.6 MJ for the group of underreporters. If this difference was mainly due to under-eating, it would probably have led to a measurable weight loss after one week. The Danish validation study tracked weight changes from start to end of the food- and activity registration period (7 days) and found a minor weight loss (-200 grams/week) [22], while others have found that underreporting was mainly caused by under-eating [4, 26].

The percentage of underreporting increased with increasing EE in our study, which is in line with a small study of young adults in Australia, comparing EI from 7-days records with DLW [27]. They found a strong correlation between increasing EE and increasing misreporting, but no association between BMI or body fat and misreporting. They concluded that people with high activity level report EI less accurately and should not be included in studies where it is important to get accurate measures of EI [27]. Results from the Australian study showed an average percentage underreporting of 22.7% and 28.8% with diet history and food records respectively, when compared with DLW.

In the present study, the group with the highest EE underreported EI by around 20 percent. This finding was not explained by higher BMI, as the percentage underreporting did not differ between normal weight and overweight participants within the group with high EE. This could be explained by a larger workload for the individuals who exercise more and/or eat more; there is more food to report and thus more food to forget [27, 28]. Another possible explanation is that larger portion sizes may be more likely of being underestimated [29]. The picture booklet with portion sizes had maximum four photos per food item or dish, of which the largest portion could be too small for a person with large food intake. It is possible to record more than one portion at a time, but we do not know how good the adult population is at choosing the closest portion when the portion eaten does not match one of the pictures. The picture booklet itself is only validated among children and adolescents, with good results [30].

According to a review article by Livingstone and Black, weight status was the single factor most strongly related to underreporting [23]. This poses a growing challenge for nutritional research, as the proportion of people being overweight or obese increases worldwide [31]. In our study, the association between high BMI and a larger percentage underreporting was only found among participants with low EE. The group with low EE and high BMI was small (n = 12), but the difference in percentage underreporting between normal weight and overweight participants was statistically significant. Thus, according to our findings, both high EE and high BMI were associated with a larger degree of underreporting and should be addressed in future validation studies comparing recorded EI to measured EE.

During the last 20 years, the PFD has been used to assess current dietary intake or changes in dietary intake in large and small studies in Norway [8, 10, 12–14]. For studies using this tool, it is essential to discuss the impact of the findings of the present validation study on their research findings. In particularly, researchers should pay attention to persons with high EE and/or high BMI, as they are at higher risk of EI underreporting.

### Strengths and limitations

The group of participants in this validation study consisted of a convenience sample and is not necessarily representative of the general adult population in Norway. Participants were recruited mostly from gyms, the military and from institutions of higher education making it probable that the participants were more active, more health conscious and more educated than the average of the population. A more representative study population, with a more moderate EE, would probably have resulted in less underreporting as the degree of underreporting increased with EE.

The recruitment of the participants was demanding and time-consuming. This was probably due to the comprehensive study design, which also included 7-days weighed record as a second part of the validation study [18]. It was particularly challenging to recruit male participants. Offering the participants some compensation might have eased the process of recruitment. Furthermore, a 2 x 4 days registration of diet would have decreased the workload and could have been sufficient to validate the PFD. The strengths of this study include the relatively large sample size and the objective measure of EE. The ActiReg system has shown good agreement when validated against DLW, is relatively cheap to administer and does not interfere greatly with daily life activities. Measured BMR would have provided a more precise calculation of EE than BMR based on measured height and weight as was done in our study, but this would have increased the workload for the study subjects.

The PFD is likely to minimize recall errors compared to retrospective methods, and it is easier and less time-consuming to complete and process compared to the weighed food record. Furthermore, the open-ended alternatives make the tool more flexible than the more commonly used food frequency questionnaire. A disadvantage of prospective dietary assessment methods, in general, is the risk of influencing the participant’s food intake (reactivity). Also, the PFD is less precise than weighted records when it comes to estimating constituents of mixed dishes, as the ingredients of mixed dishes are pre-coded.

## Conclusion

In summary, the PFD underreported EI by 17% compared to EE measured with the ActiReg in a group of motivated adults in Norway. This finding is in line with what similar validation studies of prospective dietary assessment methods have found previously. The degree of underreporting increased with increasing EE, also when we stratified by BMI. Thus the PFD seems to be less useful in groups of persons with high EE. The PFD showed a moderate ability to rank individuals according to EI.
